# Prediction of very early subclinical rejection with machine learning in kidney transplantation

**DOI:** 10.1038/s41598-023-50066-8

**Published:** 2023-12-16

**Authors:** Sung Jun Jo, Jae Berm Park, Kyo Won Lee

**Affiliations:** Department of Surgery, Samsung Medical Center, Sungkyunkwan University School of Medicine, #81 Irwon-Ro, Gangnam-Gu, Seoul, 06351 South Korea

**Keywords:** Outcomes research, Risk factors

## Abstract

Protocol biopsy is a reliable method for assessing allografts status after kidney transplantation (KT). However, due to the risk of complications, it is necessary to establish indications and selectively perform protocol biopsies by classifying the high-risk group for early subclinical rejection (SCR). Therefore, the purpose of this study is to analyze the incidence and risk factors of early SCR (within 2 weeks) and develop a prediction model using machine learning. Patients who underwent KT at Samsung Medical Center from January 2005 to December 2020 were investigated. The incidence of SCR was investigated and risk factors were analyzed. For the development of prediction model, machine learning methods (random forest, elastic net, extreme gradient boosting [XGB]) and logistic regression were used and the performance between the models was evaluated. The cohorts of 987 patients were reviewed and analyzed. The incidence of SCR was 14.6%. Borderline cellular rejection (BCR) was the most common type of rejection, accounting for 61.8% of cases. In the analysis of risk factors, recipient age (OR 0.98, *p* = 0.03), donor BMI (OR 1.07, *p* = 0.02), ABO incompatibility (OR 0.15, *p* < 0.001), HLA II mismatch (two [OR 6.44, *p* < 0.001]), and ATG induction (OR 0.41, *p* < 0.001) were associated with SCR in the multivariate analysis. The logistic regression prediction model (average AUC = 0.717) and the elastic net model (average AUC = 0.712) demonstrated good performance. HLA II mismatch and induction type were consistently identified as important variables in all models. The odds ratio analysis of the logistic prediction model revealed that HLA II mismatch (OR 6.77) was a risk factor for SCR, while ATG induction (OR 0.37) was a favorable factor. Early SCR was associated with HLA II mismatches and induction agent and prediction model using machine learning demonstrates the potential to predict SCR.

## Introduction

Kidney transplantation (KT) is a highly effective treatment for patients with end-stage renal disease (ESRD), offering better quality of life compared to long-term dialysis^1,2^. While short-term graft survival rates have significantly improved over the past few decades, there is still room for improvement in terms of long-term graft survival^3,4^.

Protocol biopsy is a reliable method for evaluating allograft status within the first year following KT^5^. Acute rejection within the first year has been shown to have a negative impact on long-term graft survival^6,7^. Therefore, there is ongoing research focused on improving graft survival through early detection and treatment of subclinical rejection (SCR) using protocol biopsies^8–10^.

Previously, our research team reported the safety and feasibility of performing protocol biopsies at the 2-week^11^. However, protocol biopsy still presents several limitations, including the risk of complications such as bleeding, cost concerns, and challenges in implementation across different centers^12,13^. Considering these factors, it is crucial to establish clear indications and selectively perform protocol biopsies in high-risk groups for early SCR.

The purpose of this study is to analyze the incidence and risk factors of early SCR based on 2-week protocol biopsy data accumulated in our center^11^, and to suggest indications for protocol biopsy. In addition, using both machine learning and logistic regression, we develop risk assessment models of SCR and compare performances.

## Method

Patients who underwent KT at Samsung Medical Center from January 2005 to December 2020 were investigated. Exclusion criteria are as follows (1) Pediatric patients, (2) received spontaneous solid organ transplantation, and (3) received dual or En-bloc KT.

Both recipient and donor data on sex, Body mass index (BMI), underlying disease, pre-dialysis information, blood type, serum creatinine, donor type (living, standard criteria deceased donor [SCD], extended criteria deceased donor [ECD]), previous transplantation history, delayed graft function, and induction agent were investigated through medical records. Data on cold ischemic time (CIT), warm ischemic time (WIT), and graft weight were investigated through operation records.

SCR were determined based on pathologic reports. Pathology was performed by dedicated specialized urology pathologist in Samsung Medical Center. All biopsy cores were obtained by two week protocol biopsy and assessed using Banff 2007 classification. The procedure details of protocol biopsy were described on a previous reported paper^11^. If borderline rejection was observed in the protocol biopsy, repeated biopsy was not performed, and steroid pulse therapy was administered.

### Classification of immunologic risk and HLA mismatch

Immunologic risks were classified into 3 groups (high, intermediate, and low). The high group was defined as patients who met any of the following conditions (ABO incompatible (ABO-i), median fluorescence intensity (MFI) value higher than 2500 with donor specific antigen (DSA), cross match positivity, or flow cytometry positivity). The intermediate group was defined as patients who met MFI value lower than 2500 with DSA, or re-transplantation. The low group was defined as patients without DSA and other immunologic risk factors.

Human Leukocyte Antigen (HLA) mismatch were evaluated for classes I and II. HLA-I mismatches were evaluated for the A and B isotypes, and HLA-II mismatches were evaluated for the DR Isotype.

### Immunosuppressive protocol

Depending on the immunologic risk, de-sensitization was performed before transplantation. In the high risk group, monoclonal antibody against CD20 (Rituximab; Genetch, Inc., South San Francisco, CA, USA) at 375 mg/m^2^ or 200 mg was administrated one month before transplantation. Plasmapheresis (PP) was started on the following day, and performed 5 times, intravenous immunoglobulin (IVIG) at 400 mg/kg was administered after every PP session. The rabbit antithymocyte globulin (rATG) was used for induction agent. For ABO-incompatible patients, PP frequency depended on baseline anti-ABO titer and target titer (1:32) before transplantation. In the intermediate risk group, monoclonal antibody against CD20 was administered one month before transplantation and rATG was used for induction agent. In the low risk group, no desensitization was performed and basiliximab was usually used for induction agent.

For maintenance, all patients were treated with a triple immunosuppressive regimen (tacrolimus, myophenolate mofetil, and methylprednisolone). The details of maintenance protocol were described in a previously reported paper^11^.

### Prediction model and machine learning

In the prediction model development for predicting SCR, dependent variables were coded as binary variables (0, 1). Patients who diagnosed with no rejection in protocol biopsy were set to 0, and patients who diagnosed with rejection in protocol biopsy, including borderline rejection, were set to 1. Data resampling was performed with hold-out validation. The ratio of the training set and the test set was set to 7:3. Three commonly used machine learning methods (random forest, elastic net, extreme gradient boosting [XGB]), and logistic regression were used to train the model^14^. As important variables in logistic regression, variables selected by backward stepwise selection in multiple logistic regression and variables with high area under a receiver operating characteristics curve (AUROC) in simple logistic regression were selected and used together. For the variable importance measures in elastic net and XGB, variables were selected through repeated cross validation. In the random forest, the definitions of the variable importance measures were as follows. The first measure is computed from permuting out-of-bag (OOB) data: for each tree, the prediction error on the OOB portion of data is recorded. Then the same is done after permuting each predictor variable. The difference between the two are then averaged over all trees, and normalized by the standard deviation of the differences. If the standard deviation of the differences is equal to 0 for a variable, the division is not done.

**Figure 1:**
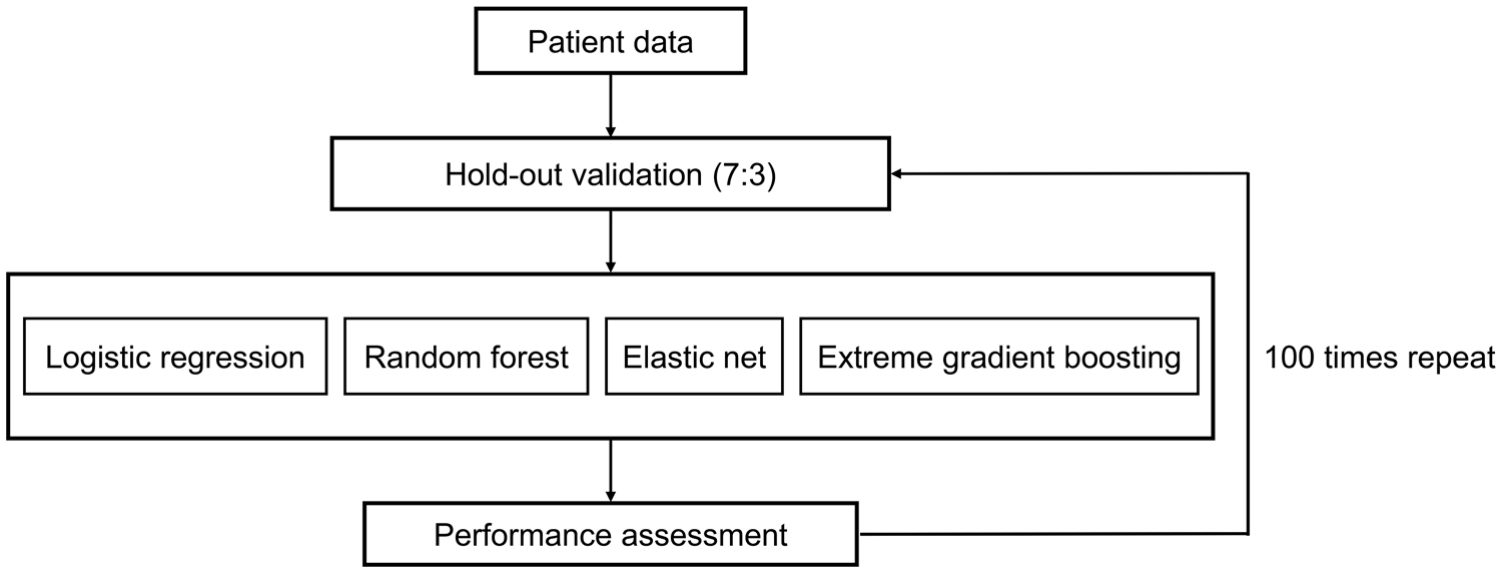
A flow diagram of developing the prediction model.

In the performance evaluation, hold-out validation was randomly repeated 100 times to build models and measure area under the curve (AUC). Model performances were evaluated using average AUC. We computed the Precision-Recall Area Under the Curve (PR AUC) for the best-performing model. Subsequently, a detailed PR analysis was conducted to derive mean and standard deviation (mean ± SD) values for precision, recall, and F1 score. Machine learning analyses were performed using R version 4.2.1, caret: Classification and Regression Training R package version 6.0-93. A flow diagram of developing the prediction model is shown in Fig. 1.

### Statistical analysis

Continuous variables with normal distribution are summarized with mean ± standard deviation, and non-normal continuous variables are expressed as the median (range). Fisher’s exact test or Pearson’s Chi-square test was applied to compare proportions between groups as appropriate. For the comparison of continuous variables, student’s t-test of Mann–Whitney *U* test were used. Logistic Regression was used to evaluate risk factor of SCR, and an estimated odd ratio (OR) with 95% confidence interval (95% CI) was presented and *p* < 0.05 was considered statistically significant. All analyses were performed using R 4.2.1 software (The R Core Team, Vienna, Austria).

### Ethical approval

The study protocol conformed to the ethical guidelines of the Declaration of Helsinki and was approved by the Institutional Review Board of Samsung Medical Center (IRB No. SMC 2023-05-157).

### Informed consent

The need for informed consent was waived by the institutional review board of Samsung Medical Center due to the retrospective nature of the study.

## Results

**Figure 2:**
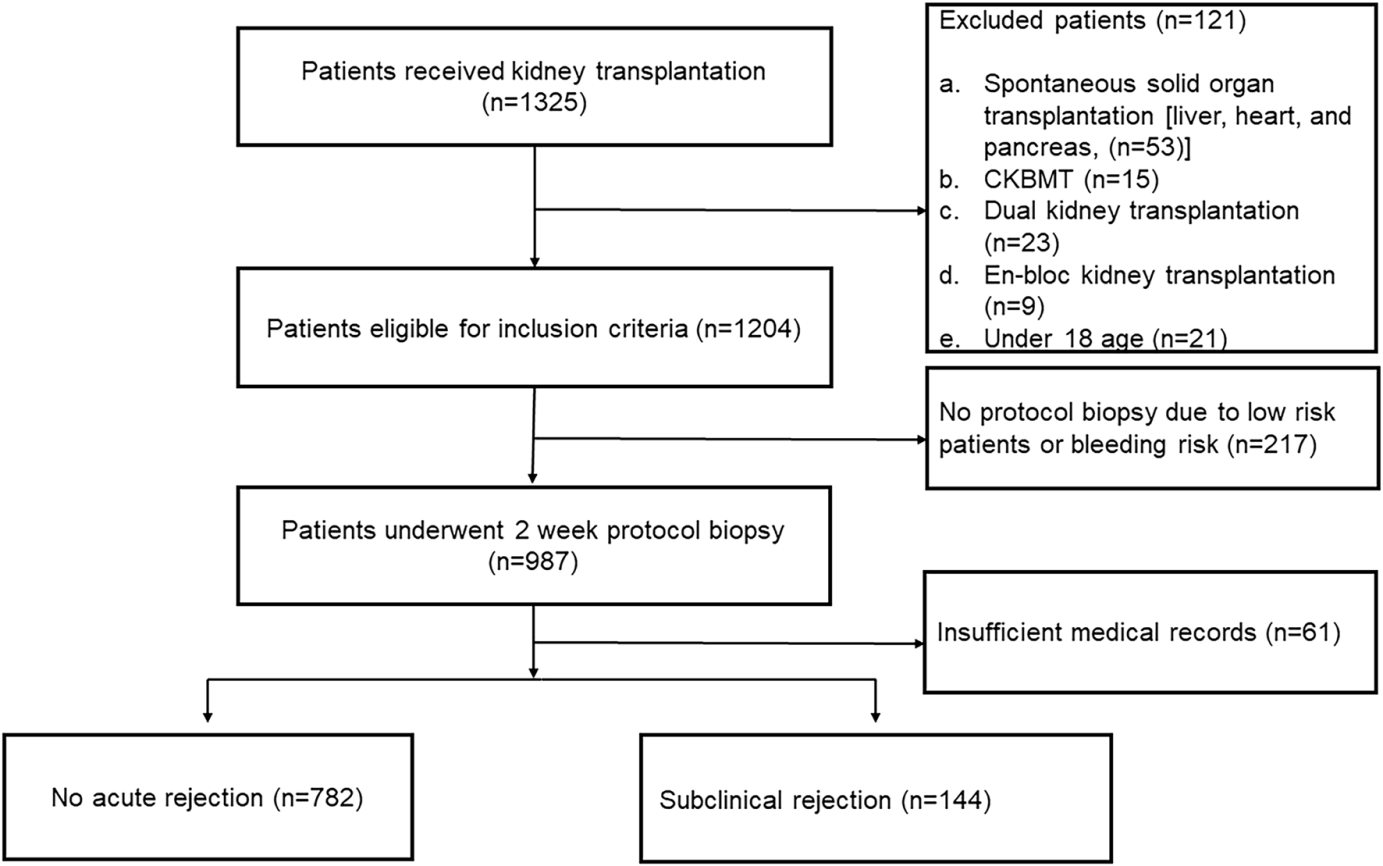
Flow diagram showing the selection criteria.

Among 1325 patients who underwent KT, 1204 were eligible for the inclusion criteria after excluding pediatric patients (n = 21), spontaneous solid organ transplantation (n = 53), dual KT (n = 23), en-bloc KT (n = 9), and combined kidney-bone marrow transplantation (CKBMT, n = 15). Two hundreds seventeen patients who could not perform a 2-week protocol biopsy due to bleeding risk and patient refusal were excluded. Sixty-one patients were excluded due to insufficient medical records. Finally, cohorts of 987 patients were reviewed and analyzed. A flow diagram showing the patients included to the study is shown in Fig. 2.

### Incidence and types of SCR

Of the total 987 patients, 144 patients demonstrated SCR. The incidence of SCR was 14.6%. The most common type of rejection was borderline cellular rejection (BCR, 61.8%), followed by acute cellular rejection (ACR, 23.6%) and acute antibody-mediated rejection (AMR, 10.4%). Mixed cellular and antibody-mediated rejection was also observed in 6 patients. The details of rejection type are summarized in Table 1.

**Table 1:** Characteristics of rejection type.

Type of rejection	Value (n, %)
Borderline cellular rejection	89 (61.8%)
Acute cellular rejection	34 (23.6%)
Acute antibody-mediated rejection	15 (10.4)
Borderline cellular rejection with antibody-mediated rejection	1 (0.7%)
Acute cellular rejection with antibody-mediated rejection	5 (3.5%)

### Comparison of characteristics between no rejection and rejection

In the recipient characteristics, the no rejection group demonstrated older than the rejection group (49.7 vs. 47.2, *p* = 0.016). There were no differences between the two groups in underlying disease, dialysis period, and underlying kidney disease. In the donor characteristics, the rejection group demonstrated a higher proportion of living donors (61.8% vs. 77.8%, *p* = 0.001) in the donor type. There were no differences in other baseline characteristics.

In the comparison of transplantation related factors, the rejection group demonstrated a higher proportion of ABO-compatible (83.4% vs. 94.4%, *p* = 0.001), and basiliximab induction (33.2% vs. 56.9%, *p* < 0.001). On the other hand, the proportion of HLA I and II zero-mismatch and immunologic high risk was lower in the rejection group. Comparison of characteristics between the no rejection group and the rejection group are summarized in Table 2.

**Table 2:** Comparison of characteristics between no rejection and subclinical rejection patients in 2 week protocol biopsy.

	No rejection (n = 782)	Rejection (n = 144)	*p* value
*Recipient characteristics*			
Age (years)	49.7 ± 11.5	47.2 ± 11.7	0.016
Sex (n, %)			
Male	475 (60.7)	88 (61.1)	
Female	307 (39.3)	56 (38.9)	1
BMI	23.3 ± 3.7	23.2 ± 3.2	0.905
Hypertension			
Yes	616 (78.8)	114 (79.2)	
No	166 (21.2)	30 (20.8)	1
Diabetes mellitus			
Yes	212 (27.1)	42 (29.2)	
No	570 (72.9)	102 (70.8)	0.684
Cardiovascular disease			
Yes	55 (7.0)	7 (4.9)	
No	727 (93.0)	137 (95.1)	0.437
Cerebrovascular disease			
Yes	14 (1.8)	5 (3.5)	
No	768 (98.2)	139 (96.5)	0.198
Pre-dialysis			
Hemodialysis	580 (74.2)	101 (70.1)	0.406
Peritoneal dialysis	53 (6.8)	14 (9.7)	
Pre-emptive	149 (19.1)	29 (20.1)	
Pre-dialysis period, month	11.5 [1.0, 69.0]	5.0 [0.0, 37.2]	0.014
Blood type			
A	244 (31.2)	56 (38.9)	0.185
B	215 (27.5)	38 (26.4)	
AB	108 (13.8)	21 (14.6)	
O	215 (27.5)	29 (20.1)	
Underlying kidney disease			
DM	187 (23.9)	37 (25.7)	0.458
Ig A	135 (17.3)	30 (20.8)	
FSGS	26 (3.3)	5 (3.5)	
GN	105 (13.4)	23 (16.0)	
Polycystic kidney disease	22 (2.8)	5 (3.5)	
Hypertension	103 (13.2)	10 (6.9)	
Others	66 (8.4)	12 (8.3)	
Unknown	138 (17.6)	22 (15.3)	
*Donor characteristics*			
Age (years)	47.9 ± 13.1	47.2 ± 13.0	0.553
Sex (n, %)			
Male	430 (55.0)	78 (54.2)	
Female	352 (45.0)	66 (45.8)	0.928
BMI	24.2 ± 3.3	24.9 ± 3.6	0.044
Hypertension			
Yes	136 (17.4)	25 (17.4)	
No	634 (81.1)	118 (81.9)	0.886
Unknown	12 (1.5)	1 (0.7)	
Diabetes mellitus			
Yes	52 (6.6)	6 (4.2)	
No	721 (92.2)	137 (95.1)	0.597
Unknown	9 (1.2)	1 (0.7)	
Blood type			
A	238 (30.4)	38 (26.4)	0.072
B	225 (28.8)	36 (25.0)	
AB	84 (10.7)	11 (7.6)	
O	235 (30.1)	59 (41.0)	
Rh type			
−	3 (0.4)	1 (0.7)	0.492
+	779 (99.6)	143 (99.3)	
Creatinine, serum	0.8 [0.7, 1.1]	0.8 [0.7, 1.0]	0.508
Estimated GFR	84.9 ± 40.9	85.1 ± 28.9	0.94
Donor type			
Living	483 (61.8)	112 (77.8)	0.001
Standard criteria deceased donor	183 (23.4)	19 (13.2)	
Extended criteria deceased donor	116 (14.8)	13 (9.0)	
*Transplantation related factors*			
ABO-incompatible			
ABO-incompatible	130 (16.6)	8 (5.6)	
ABO-compatible	652 (83.4)	136 (94.4)	0.001
Previous transplantation history			
Primary	714 (91.3)	133 (92.4)	0.895
Secondary	67 (8.6)	11 (7.6)	
Multiple	1 (0.1)	0 (0.0)	
Number of HLA I mismatch			
0	124 (15.9)	7 (4.9)	0.002
1	112 (14.3)	21 (14.6)	
2	284 (36.3)	53 (36.8)	
3	175 (22.4)	49 (34.0)	
4	87 (11.1)	14 (9.7)	
Number of HLA II mismatch			
0	219 (28.0)	14 (9.7)	< 0.001
1	399 (51.0)	70 (48.6)	
2	164 (21.0)	60 (41.7)	
Immunologic risk			
High	166 (21.2)	16 (11.1)	0.017
Intermediate	91 (11.6)	21 (14.6)	
Low	525 (67.1)	107 (74.3)	
Induction agent			
Basiliximab	260 (33.2)	82 (56.9)	< 0.001
Anti-thymocyte globulin	521 (66.6)	62 (43.1)	
None	1 (0.1)	0 (0.0)	
Warm ischemic time	31.0 [27.0, 38.0]	30.5 [26.8, 37.0]	0.112
Cold ischemic time	114.5 [76.0, 238.0]	96.5 [72.0, 144.2]	0.009
Graft weight	193.5 ± 49.4	194.2 ± 48.3	0.872
Delayed graft function			
Yes	78 (10.0)	9 (6.2)	
No	704 (90.0)	135 (93.8)	0.21

### Risk factor analyses of SCR

In the risk factor analysis of SCR, age of recipient (OR 0.98, *p* = 0.014), pre-dialysis period of recipient (OR 0.99, *p* = 0.013), donor type (SCD [OR 0.45, *p* = 0.002], ECD [OR 0.48, *p* = 0.019]), BMI of donor (OR 1.06, *p* = 0.032), ABO-i (OR 0.30, *p* = 0.001), HLA I mismatch (four [OR 2.83, *p* = 0.032]), HLA II mismatch (two [OR 5.70, *p* < 0.001], immunologic risk (intermediate [OR 2.39, *p* = 0.014], low [OR 2.12, *p* = 0.008]), and ATG induction (OR 0.38, *p* < 0.001) were statistically significant in univariate analysis. However, in the multivariate analysis, age of recipient (OR 0.98, *p* = 0.03), BMI of donor (OR 1.07, *p* = 0.02), ABO-incompatible (OR 0.15, *p* < 0.001), HLA II mismatch (two [OR 6.44, *p* < 0.001], and ATG induction (OR 0.41, *p* < 0.001) were associated with SCR (Table 3).

**Table 3:** Univariate and multivariate analyses of influencing factors associated with subclinical rejection in 2 week protocol biopsy.

Variables	n	Univariate		Multivariate	
		Crude OR (95% CI)	*p* value	Adjusted OR (95% CI)	*p* value
*Recipients characteristics*					
Age (years)		0.98 (0.97, 0.99)	0.014	0.98 (0.97, 1.00)	0.03
BMI		0.99 (0.95, 1.05)	0.916		
Hypertension, Yes (vs No)		1.03 (0.66, 1.56)	0.909		
Diabetes mellitus, Yes (vs No)		1.11 (0.75, 1.64)	0.617		
Cardiovascular disease, Yes (vs No)		0.67 (0.30, 1.51)	0.339		
Cerebrovascular disease, Yes (vs No)		1.97 (0.70, 5.56)	0.200		
Pre-dialysis					
Hemodialysis (ref.)	681				
Peritoneal dialysis	67	1.51 (0.81, 2.83)	0.194		
Pre-emptive	178	1.12 (0.71, 1.75)	0.634		
Pre-dialysis period, month		0.99 (0.99, 0.99)	0.013	1.00 (0.99, 1.00)	0.643
Blood type					
A (ref.)	300				
B	142	0.77 (0.49, 1.22)	0.265		
AB	129	0.85 (0.49, 1.47)	0.555		
O	244	0.59 (0.36, 0.95)	0.032		
Underlying kidney disease					
DM (ref.)	224				
Ig A	165	1.12 (0.66, 1.91)	0.668		
FSGS	31	0.97 (0.35, 2.70)	0.956		
GN	128	1.11 (0.62, 1.96)	0.728		
Polycystic kidney disease	27	1.15 (0.41, 3.23)	0.793		
Hypertension	113	0.49 (0.2, 1.03)	0.059		
Others	78	0.92 (0.45, 1.87)	0.815		
Unknown	160	0.81 (0.45, 1.43)	0.459		
*Donor characteristics*					
Age (years)		0.99 (0.98, 1.00)	0.547		
BMI		1.06 (1.01, 1.1)	0.032	1.07 (1.01, 1.14)	0.02
Hypertension					
No (ref.)	752				
Yes	161	0.99 (0.62, 1.58)	0.953		
Unknown	13	0.45 (0.06, 3.47)	0.441		
Diabetes mellitus					
No (ref.)	858				
Yes	58	0.61 (0.6, 1.44)	0.257		
Unknown	10	0.58 (0.07, 4.65)	0.584		
Blood type					
A (ref.)	276				
B	261	1.01 (0.62, 1.65)	0.979		
AB	95	0.82 (0.40, 1.68)	0.587		
O	294	1.57 (1.01, 2.46)	0.047		
Rh type + (vs -)		0.55 (0.06, 5.34)	0.607		
Creatinine, serum		0.91 (0.74, 1.12)	0.370		
Estimated GFR		1.00 (0.99, 1.00)	0.370		
Donor type					
Living (ref.)	595				
Standard criteria deceased donor	202	0.45 (0.27, 0.75)	0.002	0.58 (0.29, 1.17)	0.126
Extended criteria deceased donor	129	0.48 (0.26, 0.89)	0.019	0.72 (0.33, 1.55)	0.402
*Transplantation related factors*					
ABO-incompatible (vs compatible)		0.30 (0.14, 0.62)	0.001	0.15 (0.05, 0.45)	< 0.001
Previous transplantation history					
Primary (ref.)	847				
Secondary	78	0.88 (0.45, 1.71)	0.634		
Multiple	1	N/A			
Number of HLA I mismatch					
0 (ref.)	131				
1	133	3.30 (1.35, 8.05)	0.009	1.76 (0.63, 4.91)	0.278
2	337	3.28 (1.45, 7.42)	0.004	1.43 (0.55, 3.75)	0.464
3	224	4.92 (2.16, 11.23)	< 0.001	1.82 (0.66, 5.02)	0.249
4	101	2.83 (1.10, 7.30)	0.032	0.96 (0.30, 3.03)	0.94
Number of HLA II mismatch					
0 (ref.)	233				
1	469	2.73 (1.50, 4.96)	0.001	2.41 (1.20, 4.83)	0.013
2	224	5.70 (3.08, 10.55)	< 0.001	6.44 (2.98, 13.91)	< 0.001
Immunologic risk					
High (ref.)					
Intermediate		2.39 (1.19, 4.82)	0.014	0.74 (0.28, 1.95)	0.545
Low		2.12 (1.22, 3.69)	0.008	0.46 (0.19, 1.12)	0.088
Induction agent					
None	1	N/A			
Basiliximab (ref.)	342				
ATG	583	0.38 (0.26, 0.54)	< 0.001	0.41 (0.25, 0.66)	< 0.001
Warm ischemic time		1.00 (0.99, 1.00)	0.359		
Cold ischemic time		0.99 (0.99, 1.00)	0.055		
Graft weight		1.00 (0.99, 1.00)	0.895		
Delayed graft function (vs. none)		0.60 (0.29, 1.23)	0.162		

### Prediction model

**Figure 3:**
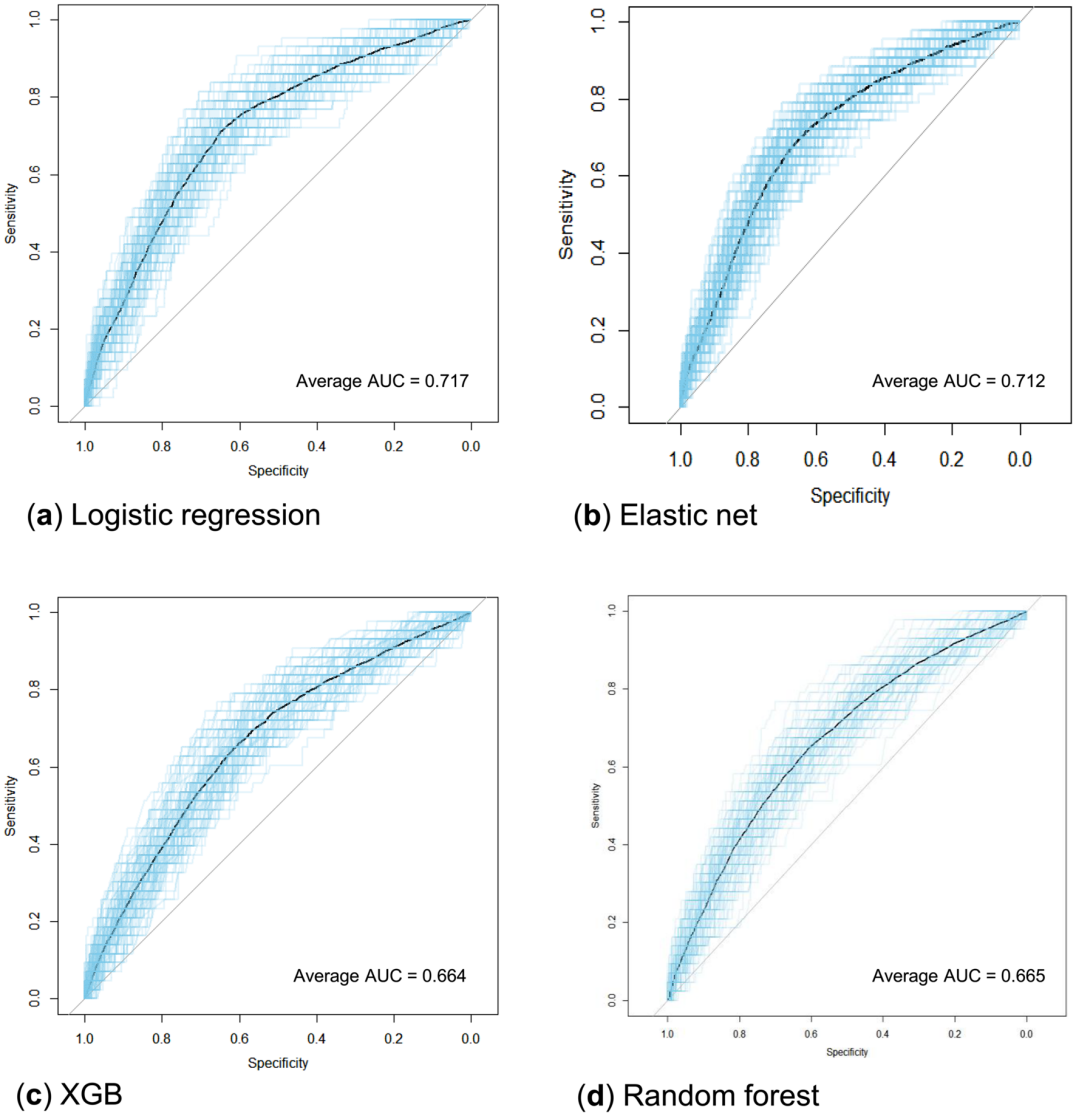
Performance evaluations of the prediction models (Average AUC of 100 times repeats). (**A**) Logistic regression. (**B**) Elastic net. (**C**) Extreme gradient boosting. (**D**) Random forest.

**Figure 4:**
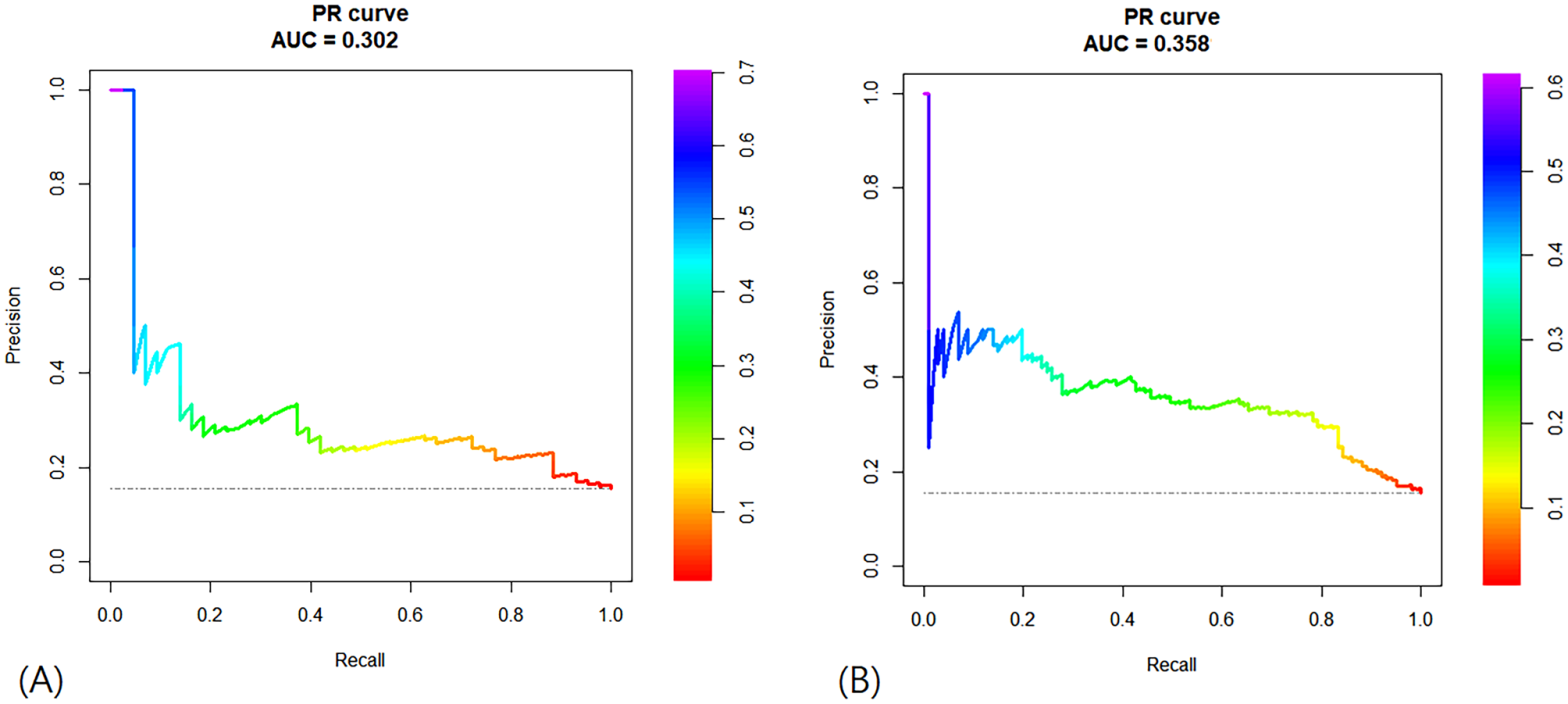
Precision-recall curve AUC of logistic regression model. (**A**) Test set. (**B**) Training set.

**Figure 5:**
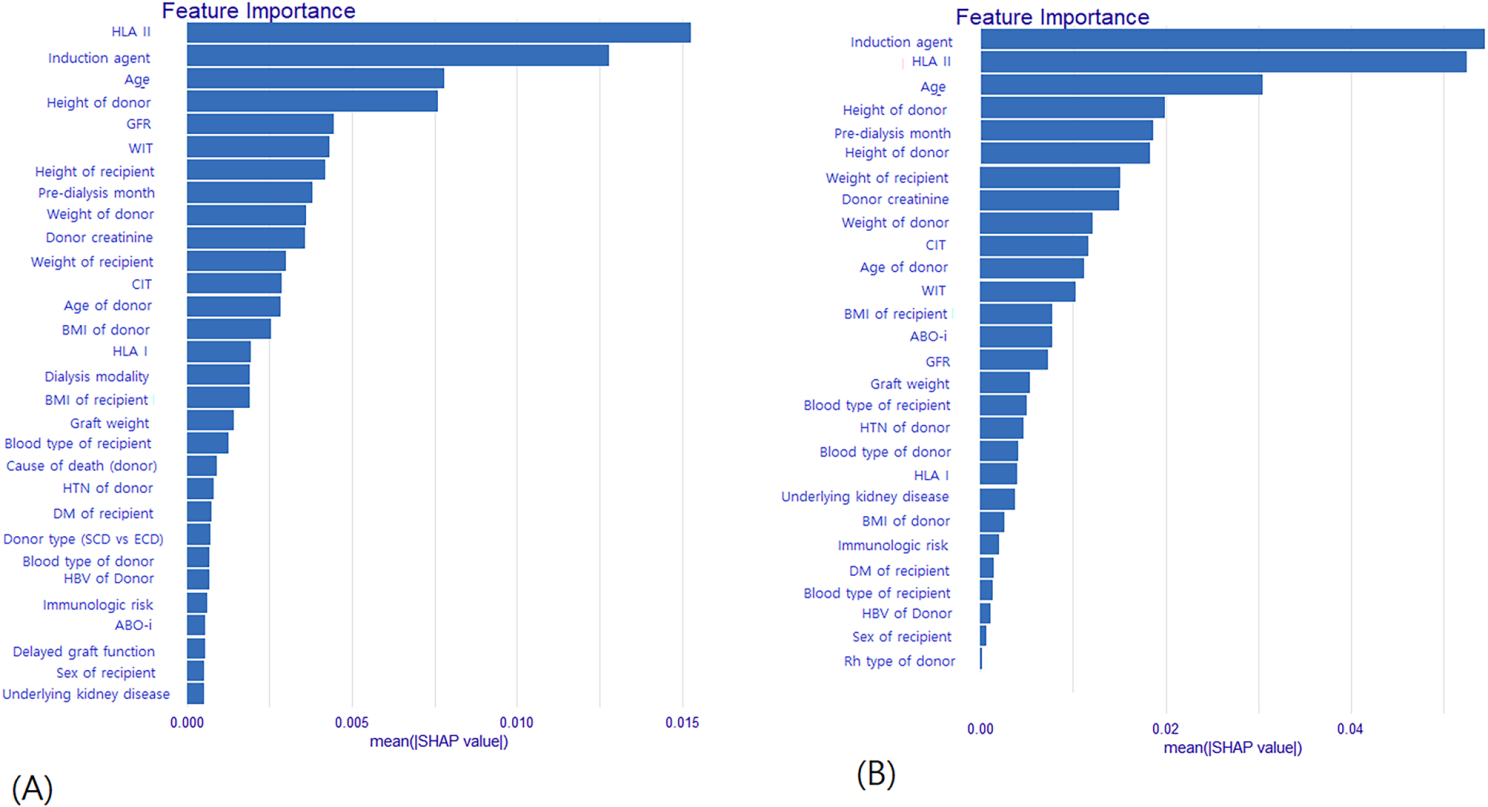
The SHAP values and important variables. (**A**) Random forest. (**B**) XGB.

The prediction model of logistic regression (average AUC = 0.717) and elastic net (average AUC = 0.712) showed good performance with an average AUC exceeding 0.7. The performance of the other two models (XGB, random forest) did not exceed an average AUC of 0.7 (Fig. 3). Additional analysis of PR AUC was conducted for the logistic regression model with the best performance. The PR-AUC for the test set was 0.302, and for the training set, it was 0.358 (Fig. 4). In the PR analysis, the logistic regression model exhibited precision of 0.143 ± 0.011, recall of 0.939 ± 0.024, and F1 score of 0.248 ± 0.016. For the random forest model, precision was 0.166 ± 0.018, recall was 0.815 ± 0.060, and F1 score was 0.275 ± 0.023. The XGB model demonstrated precision of 0.177 ± 0.024, recall of 0.689 ± 0.138, and F1 score of 0.278 ± 0.034. The variables selected in the logistic regression model were HLA II mismatch, donor BMI, induction type (Basiliximab vs. ATG), donor type (Living vs. SCD vs. ECD), and immunologic risk (high vs intermediate vs low). In the elastic net, induction type, HLA II mismatch, donor type, immunologic risk, age, recipient blood type were selected as important variables. Including analysis of variables selected from random forest and XGB, HLA II mismatch and induction type were selected as common important variables in all models. The SHAP values and important variables for XGB and random forest were demonstrated in Fig. 5. Additional OR analysis of the logistic prediction model revealed that HLA II mismatch (OR 6.77) was a risk factor for SCR, whereas ATG induction (OR 0.37) was a favorable factor.

## Discussion

KT is a therapeutic approach that significantly enhances the quality of life for patients with ESRD. However, the scarcity of organ donors poses a major challenge as the number of patients in need of transplantation far exceeds the available donors^14,15^. The occurrence of early allograft failure among KT recipients further exacerbates this mismatch, underscoring the critical importance of effective management strategies to ensure long-term graft survival.

Protocol biopsy is a technique performed at many centers for the purpose of early detection and treatment of rejection for long-term survival of allografts. Our center is also performing a protocol biopsy at 2 weeks, where safety has previously been reported^11^. However, there is a possibility of complications requiring intervention with a low probability, and there were problems in cost and time to implement in all patients. Therefore, the purpose of this study was to analyze the risk factors for SCR at 2 weeks and to make an indication for protocol biopsy.

The incidence of SCR in our study was determined to be 14.6%. When excluding BCR, the rejection rate was found to be 5.6%, which is comparable to the rejection rates (7.5–10.7%) reported in other studies involving protocol biopsies conducted within 1–6 months post-transplantation^16–18^. However, our observed rejection rate was lower than the previously reported rate of 17% for the 1–2 week period^19^.

Previous studies have indicated that HLA-A, HLA-B, and HLA-DR are potential risk factors for early SCR^19^. Oh et al.^20^ also reported that SCR was associated with HLA II mismatch and Simulet induction. Consistent with these findings, our study identified HLA II mismatch as a risk factor for early SCR. Furthermore, our results demonstrated a gradual increase in risk with an increasing number of HLA discrepancies.

In contrast to findings from other studies^21,22^, ABO-compatible KT was identified as an unfavorable factor for early SCR in our analysis. There are several potential reasons for this result. Firstly, it could be attributed to variations in the choice of induction agents. All ABO-i recipients in our study were induced with ATG, which might have influenced the outcomes. Secondly, it is plausible that the additional use of plasmapheresis in ABO-i recipients had an effect. In our center, plasmapheresis is performed if the postoperative isoagglutinin titer exceeds twice the preoperative level or if the titer does not reach 1:32 before surgery. Although plasmapheresis has been reported to reduce the occurrence of rejection after KT by eliminating preexisting antibodies, it remains unclear whether it is more effective in patients with high-sensitivity DSA or ABO-i^23,24^. Further research is warranted to investigate the specific impact of additional plasmapheresis in the context of high-sensitivity DSA and ABO-i, in order to obtain a more accurate analysis.

Machine learning represents a novel statistical approach enabling rapid analysis of complex factors and prediction of specific events. In our study, we employed the three most commonly used machine learning techniques to develop a prediction model. When comparing the performance of these models, logistic regression and elastic net demonstrated superior predictive capabilities compared to random forest and XGB. Logistic regression and elastic net are linear model-based methods, whereas random forest and XGB are tree-based models that enhance predictive accuracy by utilizing ensemble methods to estimate numerous trees. Given the observed performance differences, it is suggested that the factors influencing early SCR exhibit a linear pattern rather than a complex one.

This study was initiated to find and apply appropriate indications for protocol biopsy. Our initial hypothesis postulated a stronger association between immunologic factors, such as HLA mismatch or recipient characteristics, and the occurrence of rejection. However, the results revealed that not only HLA mismatch but also the choice of induction agent (ATG) played a significant role in the predictive model. Djamali et al. reported that the peak intensity of ATG occurs between days 6 and 8, with sustained T-cell depletion lasting beyond 20 days^25^. Taking this into consideration, it is plausible that the effect of ATG may persist during the two-week period of the protocol biopsy. Therefore, while the two-week protocol biopsy is deemed a safe procedure, it may be too early to evaluate graft function accurately due to the lingering impact of the induction agent.

This study has limitations due to the nature of a retrospective and a single center study. It is meaningful in that it showed that rejection rate that occurred very early (within 2 weeks) after KT and its risk factors. In addition, since one of the artificial intelligence (AI) technologies called machine learning was used, it showed how AI is used in the KT field. Through this result, it was found that the factors influencing the KT outcome showed a linear pattern. However, there is a limitation that these factors are selected by researchers. Therefore, in order to deduce the complex factors affecting KT through AI, future research using methods such as deep learning that can exclude human intervention is considered.

In summary, although future study is needed to determine the clinical significance of the early detection of SCR after KT at early stage, early SCR was associated with HLA II mismatches and induction agent and can be predicted by prediction model using machine learning.

## Data Availability

The data analyzed in this study are available from the corresponding author on reasonable request.
